# Evolutionary Reconstructions of the Transferrin Receptor of Caniforms Supports Canine Parvovirus Being a Re-emerged and Not a Novel Pathogen in Dogs

**DOI:** 10.1371/journal.ppat.1002666

**Published:** 2012-05-03

**Authors:** Jason T. Kaelber, Ann Demogines, Carole E. Harbison, Andrew B. Allison, Laura B. Goodman, Alicia N. Ortega, Sara L. Sawyer, Colin R. Parrish

**Affiliations:** 1 Baker Institute for Animal Health, Department of Microbiology and Immunology, College of Veterinary Medicine, Cornell University, Ithaca, New York, United States of America; 2 Section of Molecular Genetics and Microbiology, Institute for Cellular and Molecular Biology, The University of Texas at Austin, Austin, Texas, United States of America; University of California, Irvine, United States of America

## Abstract

Parvoviruses exploit transferrin receptor type-1 (TfR) for cellular entry in carnivores, and specific interactions are key to control of host range. We show that several key mutations acquired by TfR during the evolution of Caniforms (dogs and related species) modified the interactions with parvovirus capsids by reducing the level of binding. These data, along with signatures of positive selection in the *TFRC* gene, are consistent with an evolutionary arms race between the TfR of the Caniform clade and parvoviruses. As well as the modifications of amino acid sequence which modify binding, we found that a glycosylation site mutation in the TfR of dogs which provided resistance to the carnivore parvoviruses which were in circulation prior to about 1975 predates the speciation of coyotes and dogs. Because the closely-related black-backed jackal has a TfR similar to their common ancestor and lacks the glycosylation site, reconstructing this mutation into the jackal TfR shows the potency of that site in blocking binding and infection and explains the resistance of dogs until recent times. This alters our understanding of this well-known example of viral emergence by indicating that canine parvovirus emergence likely resulted from the re-adaptation of a parvovirus to the resistant receptor of a former host.

## Author Summary

Parvoviruses in cats and dogs have been studied as a model system to understand how viruses gain the ability to infect new host species. By studying the evolution of the transferrin receptor, which the virus uses to enter a cell, we discovered that the ancestors of dogs were likely infected by a parvovirus millions of years ago until they evolved and became resistant; this was caused by their transferrin receptor changing so it no longer bound the virus. When a variant virus that infects dogs emerged in the 1970s, it had adapted to overcome this block. This story suggests that diseases which were once eliminated from a species can evolve and regain the infectivity for that host, therefore having high potential to be emerging diseases. We identified features of the receptor that were important to the evolution of this host-virus interaction and confirmed their role in regulating virus binding in cell culture.

## Introduction

The interactions between viral pathogens and their hosts present a longstanding evolutionary challenge for both participants. Viruses are continuously selected for improved replication and spread in host populations, while hosts are reciprocally selected for increased resistance to infection. Thus, the viruses that exist today have been shaped by a sustained interplay with hosts over long periods of evolutionary time [1]. Much attention has been focused on the evolution of viruses, but less is known about the corresponding variation and selection of relevant host genes. However, it is clear that pathogen-driven selective pressures can also drive genetic change in the host genes that control susceptibility and disease progression. The analysis of these evolutionary interplays helps elucidate the factors that control viral emergence, defined here as the establishment of an existing virus in a novel host species.

Canine parvovirus (CPV) arose in the mid-1970s, and spread world-wide in 1978 as the cause of a new disease pandemic in dog, and that virus was clearly a variant of feline panleukopenia virus (FPV). CPV has continued to circulate among dogs throughout the world, causing significant clinical disease [2]. Parvoviruses are single-stranded DNA viruses, and the CPV- and FPV-like viruses are ubiquitous in nature, infecting most animals among the order Carnivora [3]. Viruses of the family Parvoviridae have circulated widely amongst many animal hosts for millions of years, as was revealed through the identification of ancient parvovirus genomes and genome fragments captured by vertebrate genomes tens of millions of years ago [4]–[6]. A group of parvoviruses closely related to feline panleukopenia virus (FPV) infects many hosts among the order Carnivora (carnivores), but domestic dogs, wolves, coyotes and some related carnivores resisted infection by those viruses until the emergence of CPV in 1978 [7]. The emergence of CPV in dogs was associated with the virus acquiring the ability to bind the canine transferrin receptor type-1 (TfR) [8].

While parvoviruses have certainly been evolving and changing over evolutionary time, it seems that they could also be providing a selective pressure that would conversely shape the evolution of key host genes that modulate their success, such as the *TFRC* gene that encodes TfR. Indeed, there is clear evidence of selection on host receptors in other viral-host systems, including MHC [9]; CD4 [10], and Toll-Like Receptors [11], and we wondered if the same could be true for TfR. This is important to address because viral and host controls of infection sit at the heart of our understanding of how novel viruses can emerge.

The TfR is a dimeric type II membrane protein, where each monomer is comprised of carboxypeptidase-like, helical, and apical domains, as well as stalk, transmembrane and cytoplasmic sequences. The normal function of TfR is to bind iron-loaded transferrin (Tf) via the carboxypeptidase-like and helical domains [12], [13] and mediate clathrin-mediated endocytosis. The TfR also binds to the hemochromatosis (HFE) protein which competes with the binding of Tf to regulate the uptake of iron from the intestine [14]. In our previous studies of CPV and FPV binding to the feline and canine TfR, apical domain residues were seen to be critical for controlling parvovirus binding [15], [16]. One key mutation in this domain, present in dog TfR, introduces a novel N-linked glycosylation site at Asn384 of that TfR (equivalent to feline TfR Lys383). This glycan, together with other sequence determinants in the apical domain, collectively create the block to FPV binding observed for dog cells, with the Asn384 mutation having the greatest effect [15]. As such, changing only residue 384 in the canine TfR from Asn to the feline encoded Lys allows efficient FPV binding, while the replacement in the equivalent position in the feline TfR reduces but does not eliminate FPV binding [15], [16]. However, in support of additional amino acid substitutions in the apical domain of TfR also being important, substitution of apical domain residue Leu221 in the feline TfR also reduce virus binding and cell infection by CPV and FPV [16].

Those studies raise questions about how TfR has evolved to modulate its propensity to mediate infection. However, little is know about the evolutionary history of the *TFRC* gene among animals in the order Carnivora, many of which are hosts to viruses closely related to the FPV. Two major suborders are present within the Carnivora, the Feliformia and the Caniformia, and those represent 16 families in total [17]. Confirmed or likely parvovirus infections have been reported for members of most families, although less commonly or not at all among pinnipeds [18].

Here we examined the diversity of *TFRC* gene sequences among some hosts distributed across the order Carnivora and find evidence for positive selection of this gene, more specifically in the Caniformia. Some variable sites, including some that were under positive selection, are located in the structural region of the apical domain that influences parvovirus binding. When some of these historical mutations were introduced into the apical domain of TfR they reduced binding by parvoviruses, making parvovirus a plausible selective force for the retention of these mutations when they occurred in nature. This suggests that there may have been viral pressure on this receptor before CPV emerged, some of which was exerted by ancient parvoviruses. A glycosylation site mutation present in the TfR of dogs that appears to protect dogs against FPV infection arose in a common ancestor of dogs and coyotes, suggesting that it is a recent change in that lineage.

## Results

### Acquisition of protein-altering mutations in the TfR apical domain in dogs and closely related species

Because of the recent emergence of CPV, it was previously thought that parvoviruses have been infecting Feliforms and some Caniforms for much longer than they have infected domestic dogs and closely related coyotes and wolves. We therefore wished to examine how the evolution of TfR reflected this history of infection in these different species. The sequences of the complete *TFRC* genes from 17 different carnivores, and of the apical domain from *Otocyon megalotis* (bat-eared fox), were determined by cDNA sequencing or obtained from sequence databases (Table 1). These orthologous sequences differed by up to 10% at the nucleotide level, but were easily aligned so that patterns of non-synonymous and synonymous mutational accumulation could be analyzed. The dN/dS ratio captures the number of non-synonymous mutations present per non-synonymous site (dN) compared to the number of synonymous mutations present per synonymous site (dS) [19]. Most protein-encoding genes accumulate far fewer non-synonymous mutations than synonymous mutations (dN/dS≪1) due to selective constraints [1]. In evolutionary arms race scenarios such as the ones that can develop between hosts and viruses, continued selection of each party for evolutionary dominance can cause accelerated evolution of proteins involved in the host-virus interaction. In such situations, the recurrent positive selection for non-synonymous mutations that provide a selectable advantage to each party results in relevant genes acquiring a dN/dS>1 signature [20].

**Table 1:** Host species examined in this analysis.

Common name	Scientific name	Source of sequence	Region sequenced	Accession number
Cat	*Felis catus*	[43]	Coding sequence	NM_001009312
Jungle cat	*Felis chaus*	Frozen primary culture	Coding sequence	JN887439
Pallas's cat	*Otocolobus manul*	Frozen primary culture	Coding sequence	JN887440
Asian leopard cat	*Prionailurus bengalensis*	Frozen primary culture	Coding sequence	JN887441
Cougar	*Puma concolor*	Frozen primary culture	Coding sequence	JN887442
Canada lynx	*Lynx canadensis*	Frozen primary culture	Coding sequence	JN887443
Ocelot	*Leopardus pardalis*	Frozen primary culture	Coding sequence	JN887444
Caracal	*Caracal caracal*	Frozen primary culture	Coding sequence	JN887445
Clouded leopard	*Neofelis nebulosa*	Frozen primary culture	Coding sequence	JN887446
Lion	*Panthera leo*	Frozen primary culture	Coding sequence	JN887447
Raccoon	*Procyon lotor*	Frozen tissue	Coding sequence	JN600499
Mink	*Neovison vison*	Immortalized cell line	Coding sequence	JN887448
Giant Panda	*Ailuropoda melanoleuca*	[44]	Coding sequence	NW_003217444
Red fox	*Vulpes vulpes*	Primary culture in TRIzol	Coding sequence	JN887449
Bat-eared fox	*Otocyon megalotis*	Frozen tissue	Apical domain	JN967655
Black-backed jackal	*Canis mesomelas*	Frozen tissue	Coding sequence	JN967654
Coyote	*Canis latrans*	Frozen tissue	Coding sequence	JN887450
Dog	*Canis lupus familiaris*	[8]	Coding sequence	NM_001003111
Horse	*Equus caballus*	[46]	Coding sequence	NM001081913

*TFRC* sequences were determined from cDNA prepared from mRNA isolated from the samples indicated.

**Figure 1:**
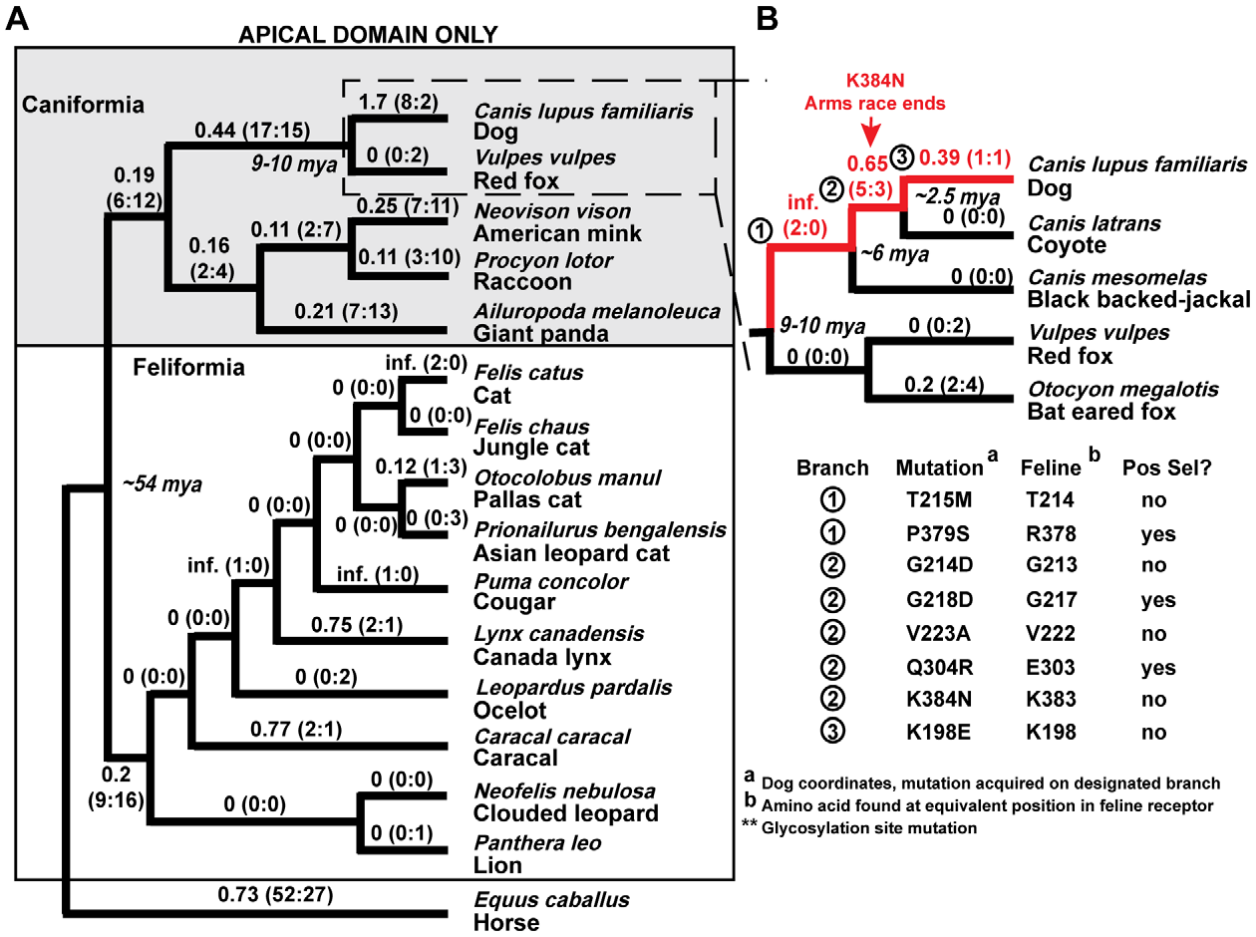
Analysis of dN/dS along each branch of the carnivore *TFRC* phylogeny. **A**) dN/dS was calculated along each branch of the carnivore phylogeny. In this analysis, only the apical domain was analyzed. The number of estimated non-synonymous and synonymous DNA mutations that have occurred along each branch are shown in parentheses (N∶S) after the dN/dS value. **B**) A secondary analysis was performed with additional canid sequences, and the relevant clade is shown. The lineage leading to dog is highlighted with red branches. Below the phylogeny, key mutations predicted to have occurred along the branches leading to dog (branches 1–3) are shown.

Because the apical domain is the binding site for parvoviruses, we first analyzed the evolutionary history of this domain by calculating the dN/dS value on each branch of the tree (Figure 1). Surprisingly, given that CPV is thought to have been in dogs for less than 40 years, the branch leading to dog has the highest value of dN/dS on the entire tree (dN/dS = 1.7). On that branch the region of *TFRC* encoding the apical domain is estimated to have accumulated 8 non-synonymous and 2 synonymous changes since dog and red fox shared their last common ancestor 9 to 10 million years ago [17], [21], [22]. In contrast, the apical domain encoding region of the red fox *TFRC* has acquired 0 non-synonymous changes and 2 synonymous changes over the same time period. To investigate this further, we examined the apical domain region of the *TFRC* genes of other Caniform species closely related to domestic dogs, the coyote, black-backed jackal, and bat-eared fox (Figure 1B). This provided increased resolution to the timing of the acquisition of the 8 non-synonymous mutations that separate the TfR apical domains of dog and red fox, showing that they accumulated along the long lineage leading to dogs (red branches labeled 1–3 in Figure 1B). The specific non-synonymous TfR mutations predicted to have occurred along key branches in the genus *Canis* (branches 1,2,3) are listed below the phylogenetic tree (Figure 1B). These include K384N (canine TfR numbering), which resulted in the novel glycosylation in the apical domain of the canine TfR which controls FPV binding [15]. Thus, the greatest rate of protein evolution observed in the apical domain occurred on the branches leading to dogs and their closest ancestors, species that are thought to have harbored parvoviruses for only around 36 years.

### Positive selection of the *TFRC* gene in carnivores and in Caniforms

The accelerated accumulation of non-synonymous mutations on the lineage leading to dogs could simply reflect relaxed selection on the apical domain of TfR in those species. In order to test the hypothesis that *TFRC* has evolved under positive selection for non-synonymous mutations, the sequences were fit to models that both allow and disallow some codons to have evolved under positive selection (dN/dS>1) using PAML [23], [24]. Null models were rejected in favor of models of positive selection in an analysis of all full-length carnivore sequences, both with and without the horse *TFRC* as an outgroup sequence (p<0.001 for both analyses; Table 2). In these analyses, between 9 and 14% of codon sites were assigned to a dN/dS class of approximately 2.0, indicating that non-synonymous mutations have been fixed at a rate approximately twice that of synonymous mutations at these codon sites. These rapidly evolving codon positions are listed in the final column of Table 2.

**Table 2:** PAML analysis of the carnivore *TFRC* gene.

Dataset^a^	2Δl^b^	p-value^b^	dN/dS^c^	% sites^c^	Tree length^d^	dN/dS>1 Codons^e^
Caniform+Feliform	13.8	p<0.001	1.9	14%	0.79	56H, 111G**, 112T, 120T, 124F**, 145S**, 150T**, 155W**, 181R, 184E**, 185F**, 190S**, 207Q**, 216E, 218D**, 301V, 304R**, 379S**, 381K**, 411R, 448L**, 502S**, 582L**, 585N, 587N**, 588Q**, 635M**, 733K
Caniform, Feliform, +Horse outgroup	20.8	p<0.001	2.5	9%	1.1	145S, 155W, 190S, 304R, 379S**, 448L, 502S, 582L**, 585N, 587N
Caniform+Horse outgroup	26.8	p<0.001	4.0	5%	0.92	145S, 190S, 304R, 379S, 582L, 585N, 587N
Feliform+Horse outgroup	1.4	p = 0.23	-	-	0.55	-

a) Datasets consisted of the aligned sequences of *Felis catus, Felis chaus, Otocolobus manul, Puma concolor, Lynx canadensis, Leopardus pardalis, Caracal caracal, Neofelis nebulosa, Prionailurus bengalensis, Panthera leo, Canis lupus familiaris, Canis latrans, Canis mesomelas, Vulpes vulpes, Neovison vison, Procyon lotor, and Ailuropoda melanoleuca*, or the indicated subset of these. In some cases, the sequence from horse (*Equus caballus*) was used as an outgroup.

b) Twice the difference in the natural logs of the likelihoods (2Δl) of the two models (M8a-M8) being compared. The p-value indicates the confidence with which the null model (M8a) can be rejected in favor of the model of positive selection (M8).

c) dN/dS value of the class of codons evolving under positive selection in M8, and the percent of codons falling in that class.

d) The tree length is the number of substitutions per site along all branches in the phylogeny. It is calculated as the sum of the branch lengths, and is a representation of total diversity in the dataset.

e) Codons assigned to the class evolving under positive selection in M8 with a posterior probability >0.95 by naive empirical Bayes (NEB) analysis (** p>0.99). Coordinates correspond to the dog protein.

We then analyzed the evolution of *TFRC* sequences from Feliform and Caniform species separately. In an analysis of the Caniform *TFRC* sequences (7 species), the null model could be rejected in favor of a model of positive selection (p<0.001; Table 2). Interestingly, although fewer codon sites were identified as evolving under positive selection (only 5% of sites), those had a higher dN/dS value (4.0) than when Feliforms had previously been included in the analysis. When Feliform *TFRC* sequences were analyzed separately, the null model of could not be rejected in favor of a model of positive selection (p = 0.23; Table 2). Even though more Feliform sequences were analyzed than in the Caniforms-only analysis (10 versus 7 species), the Feliform species analyzed are less diverged from each other and thus the tree length of this dataset was only 0.55 (Table 2). The optimal tree length for PAML analysis has been shown to be ∼1 [25], so the lack of support for positive selection in this group must be considered with that in mind. To formally test the hypothesis of Caniform-specific positive selection, we analyzed our full dataset with a “branch-site” model of evolution [26] to determine if there are codon positions evolving under positive selection specifically in the Caniform clade. This analysis supported caniform-specific positive selection (p<0.006, Table S1), consistent with the higher dN/dS value for the class of positively selected codons that was observed when Feliforms were removed in the previous analysis. These data show that *TFRC* has evolved under positive selection during the speciation of Caniforms, particularly in species closely related to modern dogs.

### Reconstructed evolutionary adaptations at positively selected sites in the TfR apical domain support an ancient arms race between parvoviruses and TfR

**Figure 2:**
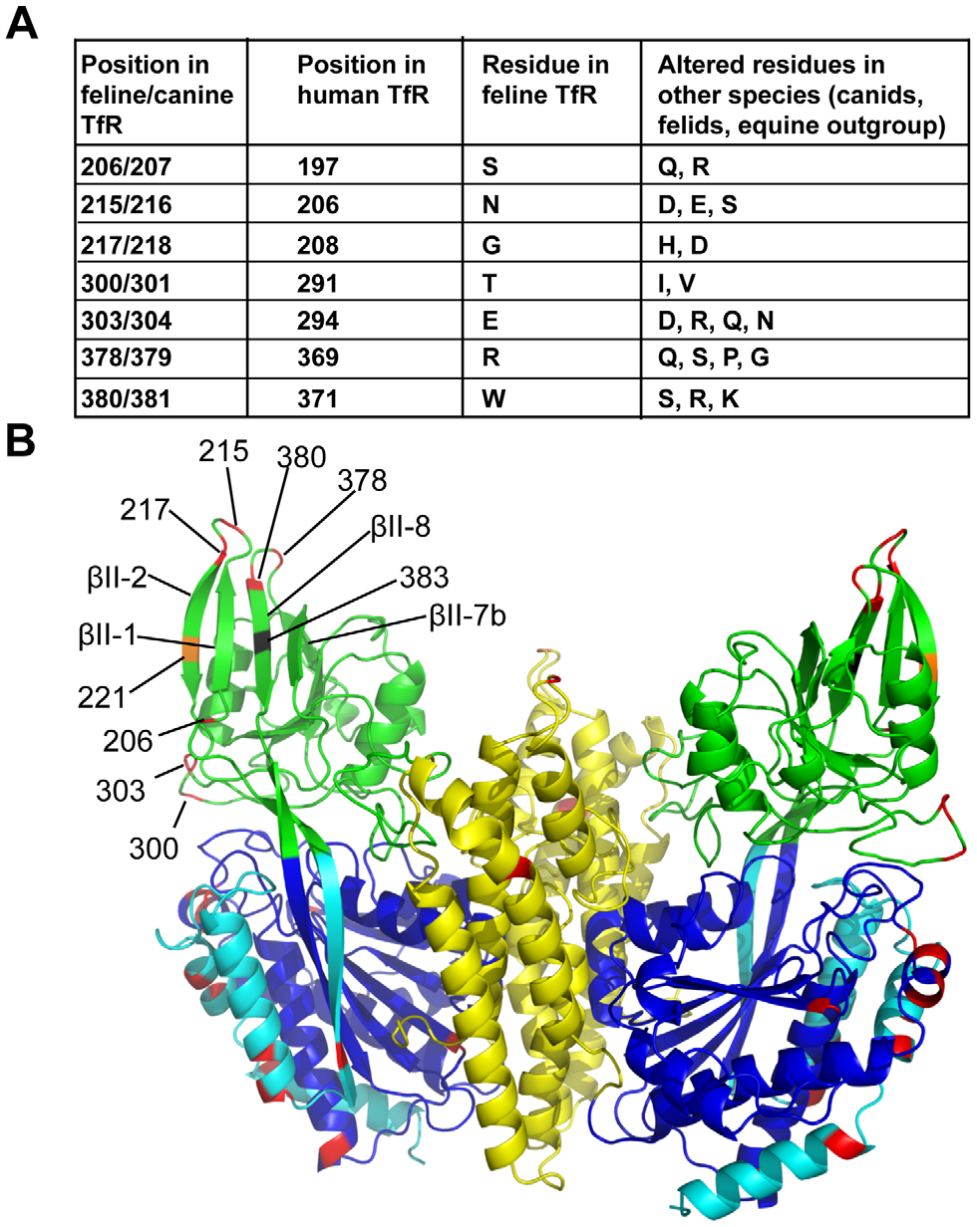
The positions of the positively selected residues in the TfR structure. **A**) A listing of the TfR residues in the apical domain under positive selection as revealed by PAML analysis, showing the positions in the different TfRs, and the alternative residues found. **B**) Residues found to be under positive selection mapped in red onto the crystal structure of the human TfR ectodomain, and those in the apical domain were labeled with the corresponding feline TfR coordinates [28]. The 3 domains of the ectodomain are shown in green (apical domain), blue (protease-like domain), and yellow (helical domain). Strands of the apical domain β-sheet which influence virus binding are labeled. Some residues under positive selection are close to the host-range determinant (feline 383/canine 384) (shown in black) and the leucine at residue 221 (shown in orange) which can be mutated to block capsid attachment, and to discriminate between FPV and CPV *in vitro* [15], [16].

We next wished to test whether the variable sites in TfR affected parvovirus binding, and therefore whether ancestors of these viruses could have been responsible for driving a least some of the rapid evolution observed in TfR. Many positively selected codons were identified in the *TFRC* sequences (Table 2), and they were mapped onto the crystal structure of the human TfR [27], [28], [29] (Figure 2). We found seven residues under positive selection in the apical domain, which is the primary binding site for FPV and CPV (Figure 2A). Interestingly, of those seven residues, three (379S, 218D, 304R (canine numbering)) have experienced a non-synonymous mutation on the lineage leading to dog during the 9 to 10 million years since the last common ancestor of fox and dogs (Figure 1B).

Sites of positive selection are also identified on other parts of the protein, which may be the result of selection by other pathogens, extant or extinct. Indeed, in rodents, two distinct viruses, New World arenaviruses and the retrovirus MMTV, bind the TfR receptor on distinct interaction interfaces [28], [30], [31]. Outside the apical domain, three features were observed which contained residues under positive selection (Figure S1). First, several residues of the stalk region were under selection in Caniformia; O-linked glycosylation of this region regulates proteolytic cleavage of the stalk and release of a soluble ectodomain [32], [33]. Second, the αI-3 helix, whose function is not yet known, contains a cluster of selected residues. Third, the αII-9 helix contains four residues under selection; mutation of this helix in a previous study reduced FPV infection but not binding [16]. The αII-9 helix lies under a disordered apical domain loop which has been implicated in parvovirus binding, and future structural studies could reveal whether these residues could be involved in binding to this virus. Signatures of positive selection were also detected in other, isolated residues. For example, a methionine at residue 635 in the helical domain is near residues involved in Tf and HFE binding, and canine residue 150 aligns next to human polymorphism S142G which is associated with type 2 diabetes [34], [35]. Further studies would be required to assign a function to any residues under positive selection outside the apical domain.

To address the possibility that an ancient arms race with parvoviruses has been responsible for the positive selection of *TFRC*, we tested whether the changes of the positively selected residues in the apical domain of TfR alter parvovirus binding. Three of the seven sites of positive selection are located in surface-exposed loop regions near the border of the apical and protease-like domains. One of these (T300; feline coordinate) was mutated in a previous study and reduced parvovirus binding and infection [16]. Four of the positively selected residues are located within two adjacent β-turns comprising the lateral tip of the apical domain, the βII-1 to βII-2 turn and the βII-7b to βII-8 turn (Figure 2B). In previous studies, residues of the βII-1 to βII-2 turn were mutated to alanines with no effect [15], [16]. Two sites under positive selection are located in or adjacent to the βII-7b to βII-8 turn. This turn is also close to the glycosylation site mutation (residue 384 in the canine TfR (383 in the feline TfR); black in Figure 2B), a critical determinant in controlling specific binding of FPV or CPV capsids. When this turn was mutated previously, many of the changes prevented cell-surface expression [15], [16]. Because no information existed on the βII-7b to βII-8 turn, we focused our attention on this structural feature using evolutionarily-informed substitutions to solve the problem of expression.

**Figure 3:**
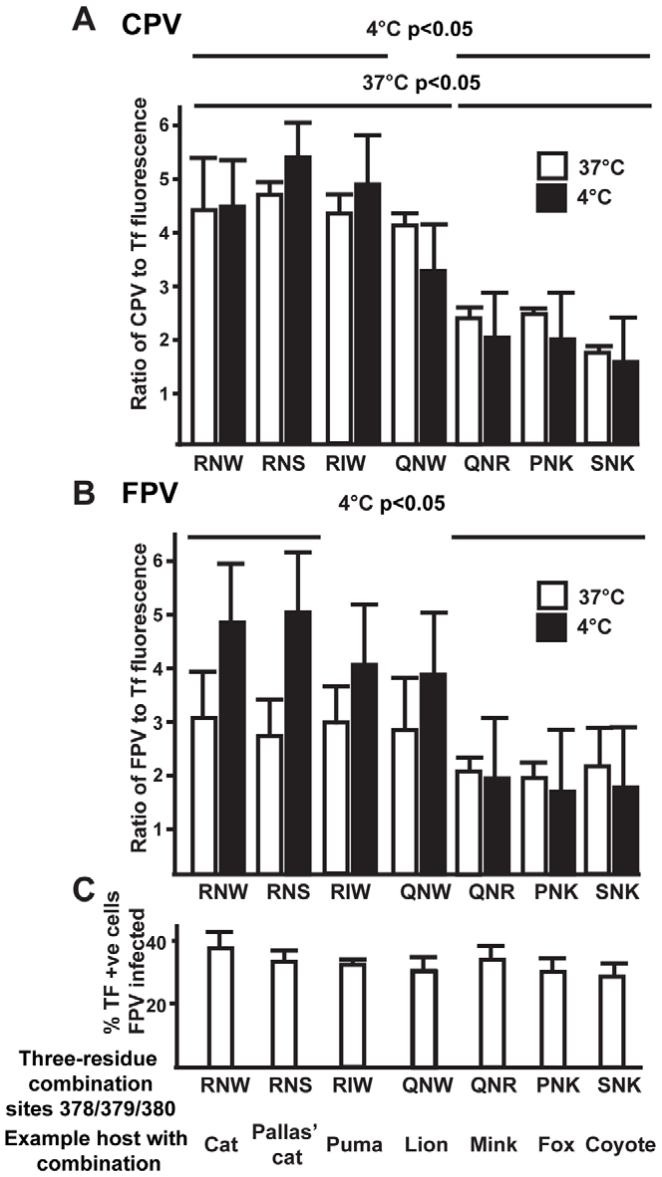
Determining the effects of varying residues in the feline TfR apical domain on parvovirus binding. CPV (**A**) or FPV (**B**) and Tf were incubated with TRVb cells expressing receptors with different combinations of the residues 378, 379, and 380 (feline TfR numbering). The name of one host species which contains the combination of residues shown is also given. Ligands were incubated with the cells at 37°C (white) or 4°C (black). Fluorescence of the labeled capsid was divided by fluorescence of the bound Tf to account for differential receptor expression. The mean of this ratio among all receptor-expressing cells was evaluated for each of three trials. The mean and standard deviation of the three trials is shown. Brackets connect groups of receptors that were statistically different in pairwise comparisons by Tukey's HSD at α = 0.05; i.e. samples not covered by a bracket did not differ at the α = 0.05 level in any comparisons. **C**) Effects of variant residues in the feline TfR on FPV infection. Cells expressing exogenous TfR with different three-amino acid combinations at residues 378–380 were inoculated and the ratio of infected cells (expressing NS1) and those expressing TfR is shown. The expressed receptors were compared for infection percentage by fitting a generalized linear mixed model to the binomial data and considering replication as a random effect, and those differed at the p = 0.034 level. Only one pairwise comparison was statistically significant after controlling for multiple testing by the Tukey-Kramer method: 34% of cells expressing TfR containing QNR (as seen in the mink TfR) were infected by FPV, while 26% of cells containing RNS (as Pallas' cat) were infected.

To test the functional affect of the observed mutations, mutations were made in the background of the feline TfR since this receptor can be utilized by both FPV and CPV [8], [36], so it can serve as a platform for testing Caniform-specific evolutionary adaptations. We mutated three residues within the βII-7b to βII-8 turn in the feline TfR, as predicted from homology modeling of the feline receptor onto the human TfR structure (positions 378, 379, and 380 (feline numbering)) to each three-residue combination found among the carnivore species for which we had sequences (Figure 3). Some of these recapitulate key mutations that were acquired by lineages in Feliformia (Pallas cat, puma, and lion), while others were mutations acquired in lineages in Caniformia (mink, fox, and coyote) (Figure 3). After expressing each mutant TfR on TRVb cells, they were tested for binding of CPV and FPV (measured at 4°C), or for binding and uptake (measured at 37°C) (Figure 3A and B). In a previous study in CHO cells (from which TRVb are derived), it was shown that holding cells at 4°C inhibited uptake in an assay modeling virus-cell fusion [37]. An antibody against the conserved cytoplasmic tail of the receptor was used to verify that each mutant TfR expressed to similar levels (Figure S2), and Tf binding was not significantly different between the various mutant TfRs (results not shown). Interestingly, several of the mutational combinations tested showed reduced parvovirus binding and uptake, consistent with the idea that naturally occurring mutations could have been selected for this purpose (Figure 3A and B). Mutations that represented combinations from Caniform species had bigger effects on binding to both viruses than those from other Feliform species.

Importantly, this indicates that there are effects from mutational differences other than the glycosylation site (Asn 384 in canine TfR) that distinguish the Feliform and Caniform TfRs with regard to virus interactions. Caniform-specific mutations reduced binding, consistent with these mutations providing an adaptive advantage against virus infection. However, the binding patterns seen were similar for both CPV and FPV, indicating that these two viruses interact with a common region of the receptor, although this is perhaps not surprising given the these two viruses are >99% identical in sequence [38]. We also tested for FPV infection of cells expressing these mutant receptors, and only small differences were observed, from a minimum of 26% to a maximum of 34% (Figure 3C), and the biological relevance of the differences is unclear. However, an ancient arms race between TfR and parvoviruses is also supported by the observation that the complementary region in the parvovirus capsid associated with receptor binding shows strong evidence of positive selection [38], [39].

### A glycosylation site in the TfR apical domain potently inhibited infection by FPV-like viruses

**Figure 4:**
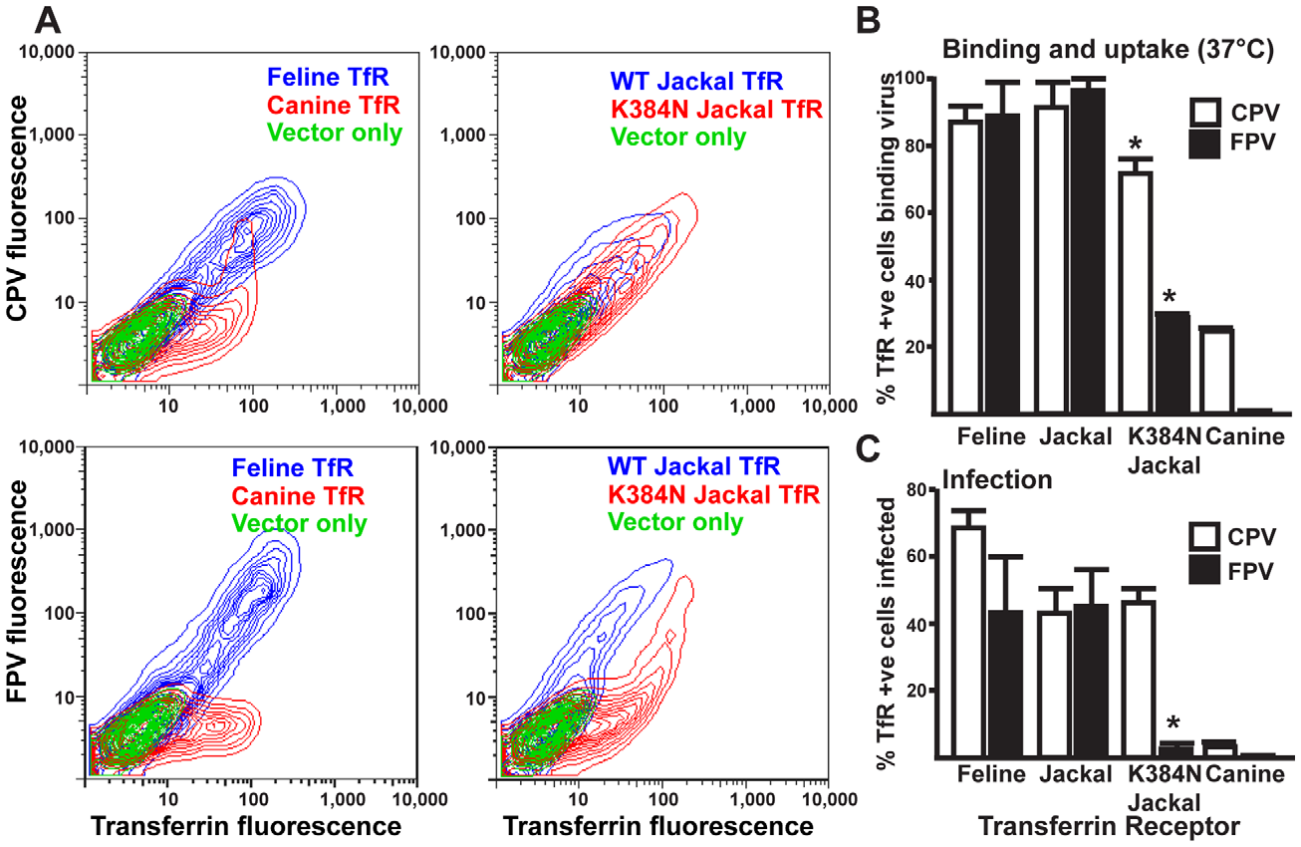
Effect of Lys or Asn at position 384 in the black-backed jackal TfR on FPV and CPV binding and infection, compared to feline or canine TfRs under the same conditions. **A**) Fluorescently labeled CPV or FPV and Tf were bound to cells expressing empty vector, feline TfR, canine TfR, wild-type jackal TfR, or Lys384Asn mutant jackal TfR at 37°C. The binding of FPV to the 384Asn black-backed jackal TfR exhibits the profile of CPV binding for canine TfR on multiple occasions [15], [16]. **B**) Fluorescently labeled FPV or CPV capsids were incubated with cells expressing feline TfR, canine TfR, wild type or mutant black-backed jackal TfR at 37°C. The binding was compared to that of fluorescently labeled Tf. **C**) Cell expressing these receptors were inoculated with FPV or CPV, and then infection measured by staining for the parvoviral NS1 expression, and the expression of TfR determined by staining for the cytoplasmic tail of the receptor. Error bars = mean ±1SD of three replicates. The wild-type and mutant black-backed jackal receptors were compared by fitting a generalized linear mixed model to the binomial data and considering replication as a random effect; * indicates statistically significant difference in frequency of binding or infection.

The N-linked glycosylation site in the apical domain of the canine TfR (at position 384 in that sequence) is critical for preventing FPV binding and infection of canine cells and dogs [36]. Among the carnivore species surveyed, only the domestic dog and coyote *TFRC* sequences encoded an Asn at this position, and we assume that it predates their speciation. However, the *TFRC* sequence of the closely related black-backed jackal does not possess this mutation. This allowed us to present the hypothesis that this single mutation could have been potent enough to end the arms race and prevent infection of the ancestors of dogs by parvoviruses for millions of years, until the emergence of CPV in 1978 (Figure 1B). We therefore introduced the Lys to Asn change into codon 384 of the black-backed jackal TfR, which diverged just before the acquisition of this K384N and four other non-synonymous mutations found in dogs and coyotes. In a previous study in which TfR was expressed in TRVb cells, the Asn to Lys change was introduced into a wild type canine TfR background and resulted in a gel shift consistent with the loss of a glycan at this site, so the mutated jackal TfR should be glycosylated in this system [15]. As can be seen in Figure 1B, there are no mutational differences in the TfR apical domain between the black-backed jackal and the most recent common ancestor of this jackal and the domestic dog, so the jackyl sequence can be thought of as an ancestral representation of the apical domain as it existed before this glycan-introducing mutation appeared. Cells expressing wild-type jackal TfR bound both FPV and CPV capsids, and were also susceptible to infection by both viruses (Figure 4, Figure S3). When the black-backed jackal TfR with the K384N change was tested, that showed significant reductions in FPV binding, uptake, and infection compared to the wildtype jackal or the feline TfR (Figure 4). However, these levels, while low, were still higher than seen for the wildtype canine TfR (Figure 4), indicating an additional role of other sequence changes in the canine TfR in controlling binding and infection by FPV. The K384N mutation in the jackal receptor did not affect CPV as much as FPV in any of the assays, and it is known that CPV successfully compensates for this novel glycosylation in the TfR to allow infection of dog cells [15], [36].

## Discussion

Here we show that there has been significant adaptive evolution of the host *TFRC* gene over the ∼54 million years of evolution of the members of the order Carnivora, and particularly in the Caniforms. One of the suggestions of this work is that parvoviruses with properties similar to those infecting hosts now were also infecting them millions of years ago, which has implications for parvovirus in the new field of “paleovirology,” the study of ancient viruses [40]. Natural selection has sampled a number of mutations in TfR over evolutionary time, and by introducing these into the background of the feline TfR we showed that some of those likely modified parvovirus interactions when they occurred. Dogs, coyotes and wolves were not infected by the FPV-related viruses until CPV emerged in the mid-1970s, and we therefore expected that the *TFRC* orthologs from those hosts would show the lowest amount of variation in the region of the apical domain which contacts the parvovirus capsid. We were surprised to find that the lineage leading to dogs showed a relatively rapid acquisition of non-synonymous mutations, most likely during the period between about 9 and 3 million years ago [22]. One of these mutations introduced a novel glycosylation site at residue 384 in the canine TfR, and that receptor was subsequently able to resist binding and infection by the FPV-like viruses, as illustrated by the reconstruction of this evolutionary event in the background of the jackal receptor. The sequence of the black backed jackal was very close to that of the ancestor of the lineage leading to dogs, and therefore this was very similar to the event that arose in the common ancestor of these hosts. The appearance of the K384N variation in the lineage leading to dogs, wolves, and coyotes likely occurred around less than six million years ago, and that may have been a potent enough mutation to inhibit parvovirus infection and extinguish the arms race. CPV subsequently arose when an FPV-like virus acquired mutational changes that made it able to efficiently infect cells expressing TfR with the canid-specific glycosylation site [8], [15].

Glycans play an important role in the biology of many pathogen receptors, and this study yields the new perspective that these glycans might sometimes be adaptively gained during host-pathogen arms races. One idea is that post-translational modifications such as glycans may provide physical distance between pathogen and receptor, explaining why mutations that introduce new glycosylation sites can be so potent. This also modifies our previous understanding of this well-known example of viral emergence, introducing the idea that canine parvovirus was a re-adaptation of the virus to the resistant receptor of a former host.

Selection on the TfR likely resulted in both the reduced binding to FPV-like ancestors through the acquisition of non-synonymous mutations, and the complete resistance through acquisition of the novel glycosylation site mutation. That resistance was clearly overcome in the 1970s when the CPV-ancestor gained the small number of capsid mutations that re-established binding of the canine TfR, allowing the emergence of CPV as a new pandemic pathogen. Therefore, the CPV host-switching event was the re-adaptation of a pathogen that had previously infected the ancestors of dogs. One question is why CPV emerged only recently, given the length of time that dogs have apparently been resistant. The size of the dog population has increased significantly in the ∼10,000 to 20,000 years since they were domesticated, and it is possible that CPV-like viruses emerging before dog domestication would not have maintained sustained transmission up to the present.

It is difficult to connect the host evolution occurring over geological timescales with the more rapid evolution of the viruses. This parvovirus model therefore provides a particularly clear description of both the host and viral sides of a long-standing interaction. An uncertainty in all studies of this type is a lack of knowledge of the viruses or other pathogens that were responsible for the selection that occurred millions of years ago. However, integrated viral sequences in various vertebrate genomes show definitively that related ancestral parvoviruses were infecting mammals millions of years ago [4], [5], [6]. While these viral sequences are 40–60% or more diverged from modern viruses in amino acid sequence, it is plausible that ancient parvoviruses could have bound the TfR and imposed the selection seen. Could an FPV-like ancestor have imposed a sufficiently strong pressure on a host population to select for variants of this key factor involved in susceptibility to infection and disease? This appears to be likely based on the few studies that have examined the effects of FPV on wild populations, where losses of up to 90% of the young each year may be due to these infections [18], [41], [42].

After mutations reducing pathogen infection become widely distributed in a host population, the development of viral adaptation to those changes is to be expected, resulting in the development of an evolutionary arms race. For viruses this has been seen in the cases of retroviruses and the cellular factors that control their infection, and in the selection of viral-controlling immune properties of the hosts and their viral countermeasures. Although the rates of mutation of hosts and their viruses are many thousand-fold different, the complexity of the processes required to overcome the host changes may cause significant delays in the acquisition of the necessary combinations of mutations [1]. In the case examined here, it may have taken millions of years for the FPV-like viruses to overcome the virus-blocking adaptations in the canine TfR, indicating the complexities of the biological and evolutionary mechanisms involved in host shifting even when only a small number of changes in the virus are required.

## Methods and Materials

### Isolate collection and sequencing

We examined a total of 19 TfR sequences (Table 1). Of those, we determined the sequences of the *TFRC* gene open reading frame of 14 host species, as listed in Table 1. In 13 cases, RNA was isolated from frozen primary cultures of cells, from frozen tissues, or from immortalized mink cells (the CCL64 cell line) using an RNeasy kit (QIAGEN Inc., Valencia, CA). RNA was isolated from *Vulpes vulpes* (red fox) tissue which had been frozen in TRIzol by the manufacturer's protocol (Invitrogen, Carlsbad, CA). One-step RT-PCR was performed using SuperScript reverse transcriptase and Platinum *Taq* DNA polymerase (Invitrogen), using primers in the 5′ and 3′ non-translated regions of the TfR mRNA. PCR products were purified using a QIAquick kit (QIAGEN) and either directly sequenced or cloned into the plasmid pCR-2.1-TOPO, and the cloned fragment sequenced.

### Sequence analysis

In addition to the 14 sequences determined here, we analyzed previously published *Felis catus* and *Canis lupus familiaris TFRC* sequences [8], [43] as well as the Giant Panda (*Ailuropoda melanoleuca*) *TFRC* derived from the panda genome project [44]. The raccoon (*Procyon lotor*) *TFRC* sequence was obtained from a raccoon cell line and from a raccoon tissue sample [45], while the horse (*Equus caballus*) *TFRC* sequence used as an outgroup was previously published [46]. The multiple sequence alignment generated for *TFRC* was analyzed for positive selection with the “codeml” program in PAML [24]. This offers several models for gene evolution, some where no codons are allowed to evolve with dN/dS>1 (NSsites models M1a, M7 and M8a), and others where positive selection of some codons is allowed (NSsites models M2a and M8). A likelihood ratio test allows comparison of positive selection models to null models.

### Cells and receptor expression

TRVb cells (Chinese hamster ovary cells, which do not express endogenous TfR) were cultured in Ham's F12 medium with 5% fetal calf serum [47]. The black-backed jackal TfR was amplified as described above, cloned in the pCDNA3.1(−) vector for expression. The feline and canine TfR constructs used are previously described [43]. Mutations were introduced into the jackal and feline TfRs using the Phusion mutagenesis protocol, as previously described [16]. Receptor expressing plasmids were transfected into TRVb cells seeded in 9 cm^2^ trays at a density of 2×10^4^ cells per cm^2^ and transfected with 1.5 µg (for infection assays) or 2 µg (for binding assays) of TfR plasmid or pCDNA 3.1(−) using Lipofectamine (Invitrogen).

### Analysis of infection

Two days after transfection, TRVb cells were detached with trypsin/versene and seeded at a 1∶5 dilution on coverslips. The next day the cells were washed and incubated for one hour with FPV, CPV, or virus-free medium. Five days after transfection, cells were fixed in 4% paraformaldehyde and stained with mouse anti-human TfR cytoplasmic tail antibody (clone H68.4, Invitrogen) followed by Alexa 488-conjugated goat anti-mouse secondary antibody to detect TfR, and then with Alexa 594-conjugated mouse anti-NS1 antibody [48].

### Virus and Tf binding assays

Two days after transfection, TRVb cells were washed with cold Dulbecco's PBS and detached using Accutase (Innovative Cell Technologies, San Diego, CA). Cells were pelleted and washed in PBS containing 1% ovalbumin. Cells were then incubated for one hr at 37°C or at 4°C with iron-loaded canine Tf conjugated to PerCP dye, and Alexa488-conjugated genome-free CPV-2 or FPV capsid. After washing with PBS with 1% ovalbumin, 10,000 cells were analyzed by a Guava EasyCyte Plus (Millipore, Billerica, MA). Cells were gated based on forward and side scatter and compensated in FlowJo 9 (TreeStar Inc, Ashland, OR). Tf-positive cells were gated in FlowJo and exported to JMP for statistical analysis because Tf labels the transfected cells expressing the receptors. All receptors are expressed to similar levels on TRVb cells, as revealed by staining with an antibody against the cytoplasmic tail of the receptor (Figure S2). Since Tf does not compete with parvovirus for TfR binding [15], [16] and the mutations introduced are far removed from the TfR binding site, the relative Tf-PerCP fluorescence of cells reflected the expression of the receptor on that cell. For each cell, the fluorescence intensity of parvovirus binding was normalized to the fluorescence intensity of Tf. One way analysis of variance of the mean ratio of fluorescence intensities of each receptor was performed to determine the degree of parvovirus binding, or binding and uptake. Since the choice of the domestic cat TfR for this study is arbitrarily chosen as the background for the mutation analysis, Tukey's HSD was used to detect significantly different levels of binding and uptake instead of pairwise comparisons to the feline TfR.

## Supporting Information

Figure S1 Analysis of dN/dS in the apical and non-apical domains of *TFRC*. dN/dS was calculated along each branch of the carnivore phylogeny for **A**) the apical domain, and **B**) the concatenated remainder of the gene. The number of estimated non-synonymous and synonymous DNA mutations that have occurred along each branch are shown in parentheses (N∶S) after the dN/dS value. On each branch in Caniformia, we highlight in red the part of the gene with the highest dN/dS value. **C and D**) A secondary analysis was performed on additional canid sequences closely related to dogs. Both in these analyses, and in the feliformia species in panels A and B, the branches are too short (too few evolutionary changes have taken place) to draw clear conclusions.
(TIF)

Figure S2 Expression of variant TfRs in TRVb cells. Cells transfected with various TfRs were fixed, permeabilized, and stained with antibody against the conserved, cytoplasmic tail of the receptor and quantitatively analyzed by flow cytometry. Cells were gated for receptor expression and the mean fluorescent intensity of receptor-expressing cells is shown. No comparisons were statistically significant at p = 0.05.
(TIF)

Figure S3 Effect of residue 383 (feline) or 384 (canine and jackal) in the TfR on virus binding at 4°C. Fluorescently labeled FPV or CPV capsids were incubated with cells expressing feline TfR, canine TfR, wild type or mutant black-backed jackal TfR at 4°C, and the virus binding was compared to that of fluorescently labeled Tf.
(TIF)

Table S1 Branch-site test for positive selection in the Caniformia clade for the carnivore *TFRC* gene. **a.** Datasets consisted of the aligned sequences of *Canis lupus familiaris*, *Canis latrans*, *Canis mesomelas*, *Vulpes vulpes*, *Neovison vison*, *Procyon lotor*, *Ailuropoda melanoleuca*, *Felis catus*, *Felis chaus*, *Otocolobus manul*, *Puma concolor*, *Lynx canadensis*, *Leopardus pardalis*, *Caracal caracal*, *Neofelis nebulosa*, *Prionailurus bengalensis*, and *Panthera leo*, with *Equus caballus* used as an outgroup. The Caniformia clade (the first 7 species listed) is defined as the foreground clade in the models. **b.** To implement the branch-sites test (Zhang et al., 2005), multiple alignments were fit to the branch-sites models Model A (positive selection model, codon values of dN/dS along background branches are fit into two site classes, one (ω0) between 0 and 1, and one (ω1) equal to 1; on the foreground branches a third site class is allowed (ω2) with dN/dS>1), and Model A with fixed ω2 = 1 (null model, similar to Model A except the foreground ω2 value is fixed at 1). Thus, the branch-sites model for positive selection (Model A) allows certain codons to evolve with dN/dS>1 exclusively along the lineages of the foreground clade. The likelihood of this model is compared to the likelihood of the null model, where dN/dS>1 is disallowed along both foreground and background lineages. A likelihood ratio test was performed to assess whether Model A gives a significantly better fit to the data (branch-site test 2). The f61 codon model was used, and for Model A an initial seed value of ω = 1.5 was used. **c.** Twice the difference in the natural logs of the likelihoods (Δl×2) of the two models being compared. The p-value indicates the confidence with which the null model can be rejected.
(DOC)
