# Supplementary figures and images for: Evolutionary Reconstructions of the Transferrin Receptor of Caniforms Supports Canine Parvovirus Being a Re-emerged and Not a Novel Pathogen in Dogs
Source: PLoS Pathog. 2012 May 3;8(5):e1002666. doi: 10.1371/journal.ppat.1002666 (PMC3342950; doi:10.1371/journal.ppat.1002666)

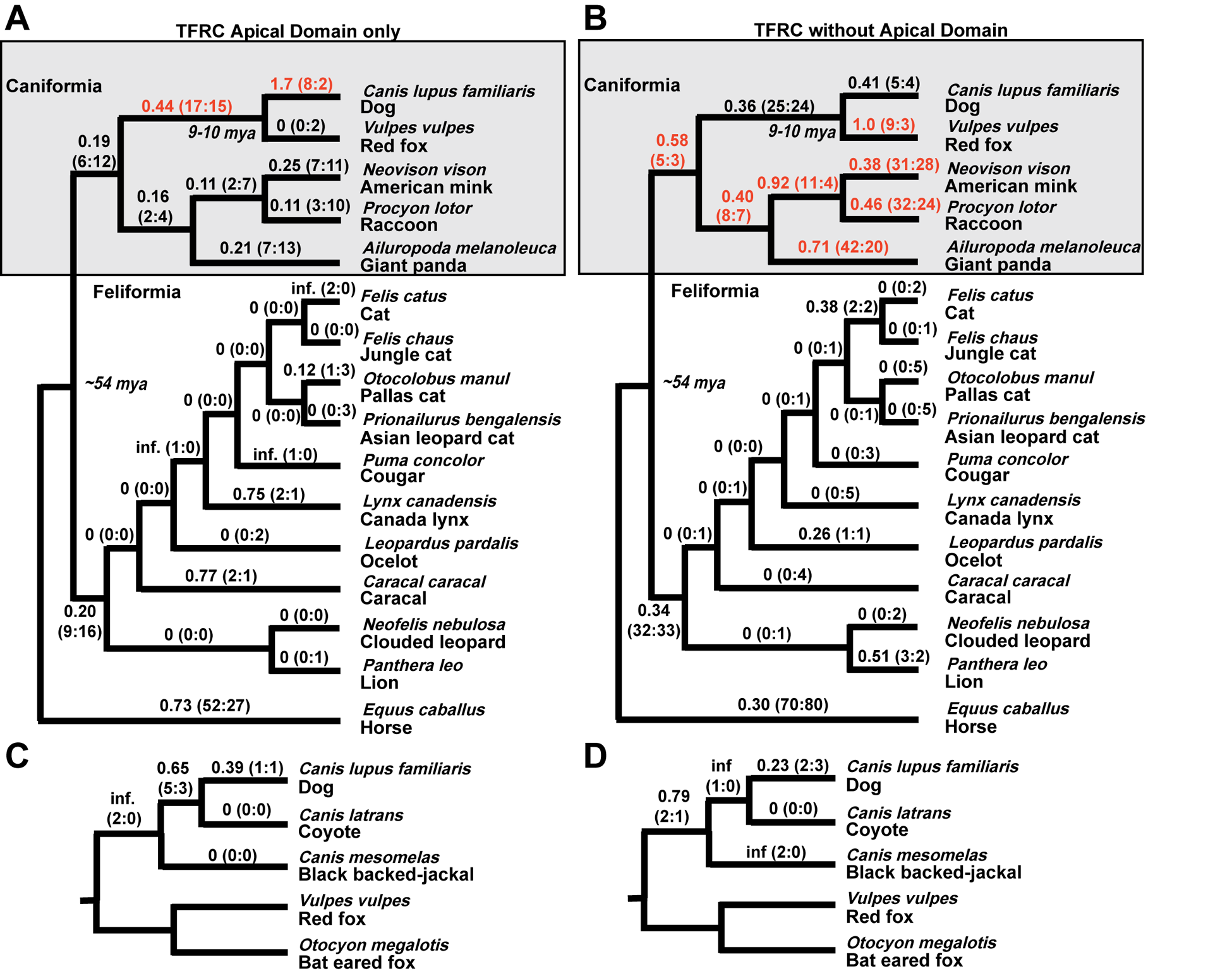

Supplement: Figure S1 — Analysis of dN/dS in the apical and non-apical domains of TFRC. dN/dS was calculated along each branch of the carnivore phylogeny for A) the apical domain, and B) the concatenated remainder of the gene. The number of estimated non-synonymous and synonymous DNA mutations that have occurred along each branch are shown in parentheses (N∶S) after the dN/dS value. On each branch in Caniformia, we highlight in red the part of the gene with the highest dN/dS value. C and D) A secondary analysis was performed on additional canid sequences closely related to dogs. Both in these analyses, and in the feliformia species in panels A and B, the branches are too short (too few evolutionary changes have taken place) to draw clear conclusions. (TIF) [file ppat.1002666.s001.tif]

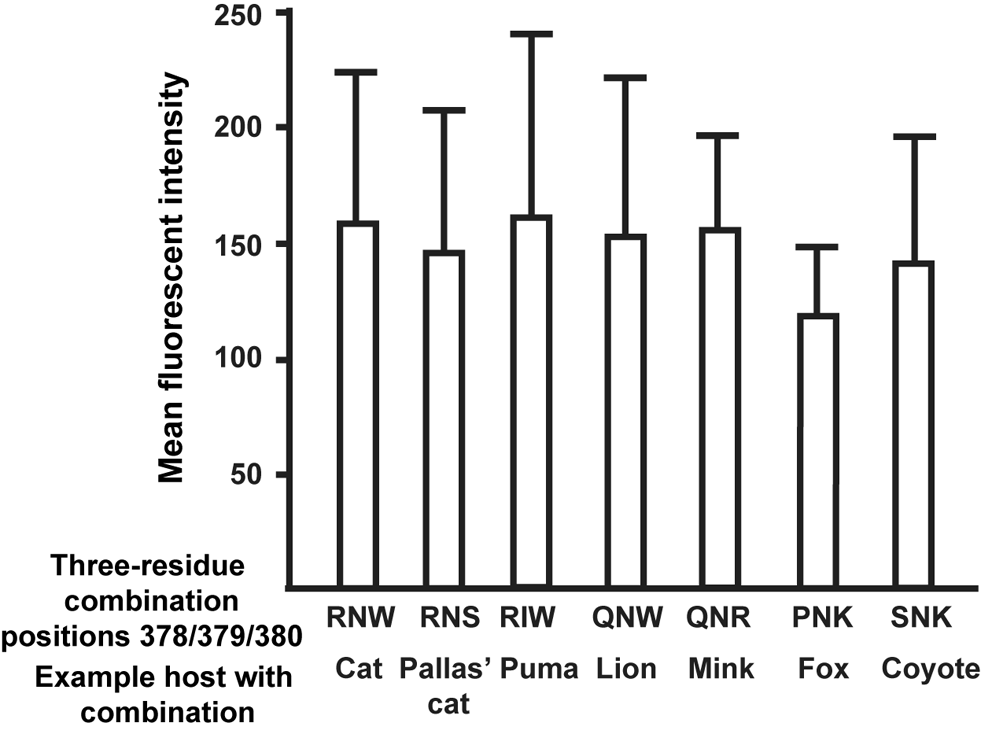

Supplement: Figure S2 — Expression of variant TfRs in TRVb cells. Cells transfected with various TfRs were fixed, permeabilized, and stained with antibody against the conserved, cytoplasmic tail of the receptor and quantitatively analyzed by flow cytometry. Cells were gated for receptor expression and the mean fluorescent intensity of receptor-expressing cells is shown. No comparisons were statistically significant at p = 0.05. (TIF) [file ppat.1002666.s002.tif]

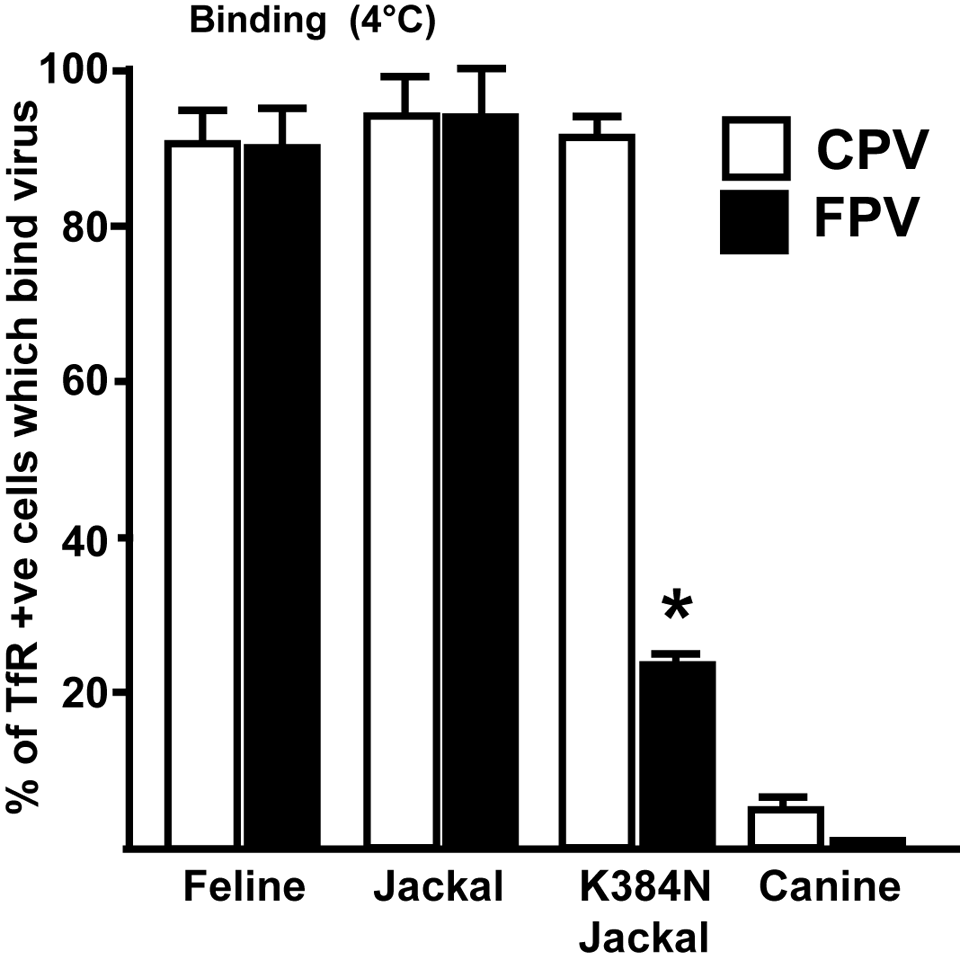

Supplement: Figure S3 — Effect of residue 383 (feline) or 384 (canine and jackal) in the TfR on virus binding at 4°C. Fluorescently labeled FPV or CPV capsids were incubated with cells expressing feline TfR, canine TfR, wild type or mutant black-backed jackal TfR at 4°C, and the virus binding was compared to that of fluorescently labeled Tf. (TIF) [file ppat.1002666.s003.tif]

**Table S1.**

**
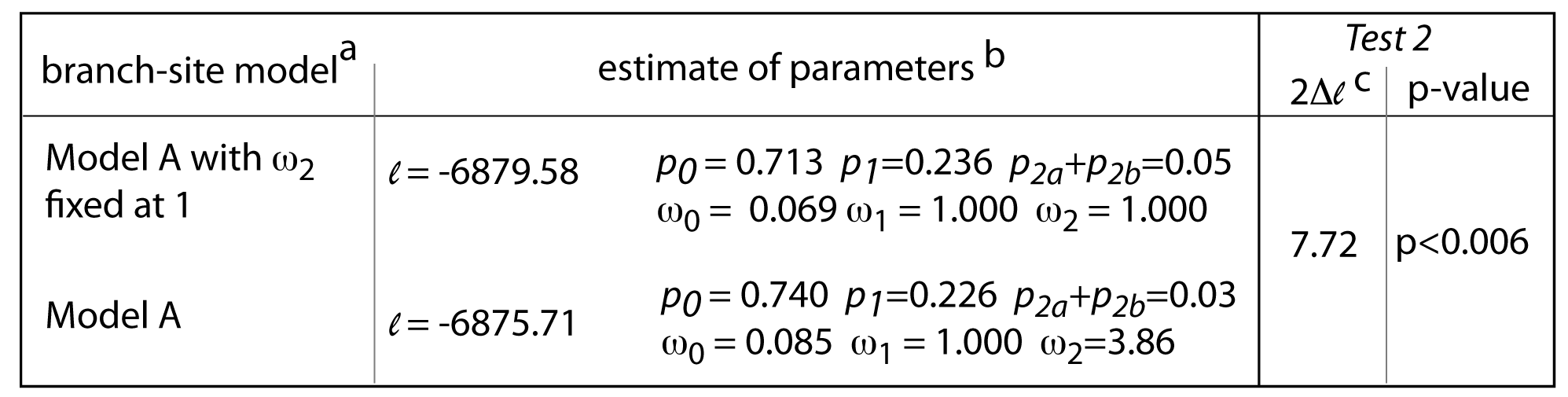
**

Supplement: Table S1 — Branch-site test for positive selection in the Caniformia clade for the carnivore TFRC gene. a. Datasets consisted of the aligned sequences of Canis lupus familiaris, Canis latrans, Canis mesomelas, Vulpes vulpes, Neovison vison, Procyon lotor, Ailuropoda melanoleuca, Felis catus, Felis chaus, Otocolobus manul, Puma concolor, Lynx canadensis, Leopardus pardalis, Caracal caracal, Neofelis nebulosa, Prionailurus bengalensis, and Panthera leo, with Equus caballus used as an outgroup. The Caniformia clade (the first 7 species listed) is defined as the foreground clade in the models. b. To implement the branch-sites test (Zhang et al., 2005), multiple alignments were fit to the branch-sites models Model A (positive selection model, codon values of dN/dS along background branches are fit into two site classes, one (ω0) between 0 and 1, and one (ω1) equal to 1; on the foreground branches a third site class is allowed (ω2) with dN/dS>1), and Model A with fixed ω2 = 1 (null model, similar to Model A except the foreground ω2 value is fixed at 1). Thus, the branch-sites model for positive selection (Model A) allows certain codons to evolve with dN/dS>1 exclusively along the lineages of the foreground clade. The likelihood of this model is compared to the likelihood of the null model, where dN/dS>1 is disallowed along both foreground and background lineages. A likelihood ratio test was performed to assess whether Model A gives a significantly better fit to the data (branch-site test 2). The f61 codon model was used, and for Model A an initial seed value of ω = 1.5 was used. c. Twice the difference in the natural logs of the likelihoods (Δl×2) of the two models being compared. The p-value indicates the confidence with which the null model can be rejected. (DOC) [file ppat.1002666.s004.doc]
